# Fluorofenidone attenuates pulmonary inflammation and fibrosis *via* inhibiting the activation of NALP3 inflammasome and IL‐1β/IL‐1R1/MyD88/NF‐κB pathway

**DOI:** 10.1111/jcmm.12898

**Published:** 2016-06-16

**Authors:** Cheng Song, Lujuan He, Jin Zhang, Hong Ma, Xiangning Yuan, Gaoyun Hu, Lijian Tao, Jian Zhang, Jie Meng

**Affiliations:** ^1^ Department of Respiratory MedicineXiangya HospitalCentral South UniversityChangshaChina; ^2^ Department of Respiratory MedicineCentral Hospital of WuhanTongji Medical College Huazhong University of Science & TechnologyWuhanChina; ^3^ Department of Nephrology MedicineXiangya HospitalCentral South UniversityChangshaChina; ^4^ Pharmaceutical SchoolCentral South UniversityChangshaChina; ^5^ Department of Microbial Infection & ImmunityThe Ohio State UniversityColumbusOHUSA

**Keywords:** fluorofenidone, pulmonary fibrosis, NALP3 inflammasome, IL‐1β/IL‐1R1/MyD88/NF‐κB pathway

## Abstract

Interleukin (IL)‐1β plays an important role in the pathogenesis of idiopathic pulmonary fibrosis. The production of IL‐1β is dependent upon caspase‐1‐containing multiprotein complexes called inflammasomes and IL‐1R1/MyD88/NF‐κB pathway. In this study, we explored whether a potential anti‐fibrotic agent fluorofenidone (FD) exerts its anti‐inflammatory and anti‐fibrotic effects through suppressing activation of NACHT, LRR and PYD domains‐containing protein 3 (NALP3) inflammasome and the IL‐1β/IL‐1R1/MyD88/NF‐κB pathway *in vivo* and *in vitro*. Male C57BL/6J mice were intratracheally injected with Bleomycin (BLM) or saline. Fluorofenidone was administered throughout the course of the experiment. Lung tissue sections were stained with haemotoxylin and eosin and Masson's trichrome. Cytokines were measured by ELISA, and α‐smooth muscle actin (α‐SMA), fibronectin, collagen I, caspase‐1, IL‐1R1, MyD88 were measured by Western blot and/or RT‐PCR. The human actue monocytic leukaemia cell line (THP‐1) were incubated with monosodium urate (MSU), with or without FD pre‐treatment. The expression of caspase‐1, IL‐1β, NALP3, apoptosis‐associated speck‐like protein containing (ASC) and pro‐caspase‐1 were measured by Western blot, the reactive oxygen species (ROS) generation was detected using the Flow Cytometry, and the interaction of NALP3 inflammasome‐associated molecules were measured by Co‐immunoprecipitation. RLE‐6TN (rat lung epithelial‐T‐antigen negative) cells were incubated with IL‐1β, with or without FD pre‐treatment. The expression of nuclear protein p65 was measured by Western blot. Results showed that FD markedly reduced the expressions of IL‐1β, IL‐6, monocyte chemotactic protein‐1 (MCP‐1), myeloperoxidase (MPO), α‐SMA, fibronectin, collagen I, caspase‐1, IL‐1R1 and MyD88 in mice lung tissues. And FD inhibited MSU‐induced the accumulation of ROS, blocked the interaction of NALP3 inflammasome‐associated molecules, decreased the level of caspase‐1 and IL‐1β in THP‐1 cells. Besides, FD inhibited IL‐1β‐induced the expression of nuclear protein p65. This study demonstrated that FD, attenuates BLM‐induced pulmonary inflammation and fibrosis in mice *via* inhibiting the activation of NALP3 inflammasome and the IL‐1β/IL‐1R1/MyD88/ NF‐κB pathway.

## Introduction

Idiopathic pulmonary fibrosis (IPF) is defined as ‘a specific form of chronic, progressive fibrosing interstitial pneumonia of unknown cause’ 1. It is characterized by massive activated fibroblasts differentiation into myofibroblasts and excess deposition of extracellular matrix (ECM) 2. In the initial injury phase, activated alveolar epithelial cells and recruited inflammatory cells release chemokines and pro‐inflammatory cytokines, leading to inflammatory cells recruitment, remodeling, and lung fibrosis 3. Tissue injury and acute inflammatory responses lead to the imbalance of proteases (matrix metalloproteinases), antiproteases (tissue inhibitors of metalloproteinases), and the activation of myofibroblasts, resulting in the increased deposition of ECM which is the characteristic of IPF 4, 5, 6.

Interleukin (IL)‐1β, a pleiotropic cytokine, is thought to play a central role in pulmonary fibrosis, can stimulate collagen expression in a dose dependent manner *in vitro* 7. Administration of recombinant mouse IL‐1β to the wild‐type mice induced significant tissue destruction with increased inflammation and collagen deposition 8. Two signalling pathways are generally required for IL‐1β production: one triggers proteolytic pro‐IL‐1β processing to produce bioactive IL‐1β *via* caspase‐1‐containing multiprotein complexes called inflammasomes, the other is NF‐κB‐dependent signalling pathway that modulates the mRNA expression of IL‐1β 9, 10. Several studies have shown that uric acid and ATP released from BLM‐injured lung cells constitute major endogenous danger signals that increase the mitochondrial reactive oxygen species (ROS) generation, and then activate the NALP3 inflammasome characterized by the maturation of pro‐caspase‐1, leading to IL‐1β production and pulmonary fibrosis 11, 12, 13, 14, 15, 16. Administration of the inhibitor of caspase‐1 z‐YVAD‐fmk or knockout caspase‐1 in mice attenuated BLM‐induced production of IL‐1β, pulmonary inflammation and fibrosis 11. Besides, IL‐1β activated the IL‐1R1/MyD88 complex in tissue‐resident cells, mainly pulmonary epithelial cells, and then activated transcription factors such as NF‐κB, leading to inflammation with neutrophil and lymphocyte recruitment and fibroblast activation 8. In mice that were IL‐1R1 deficient or MyD88 deficient, or administration of IL‐1 receptor antagonist (Anakinet) abrogated responses to BLM, such as the release of pro‐inflammatory cytokines, pulmonary inflammation and fibrosis 8.

Fluorofenidone [1‐(3‐fluorophenyl)‐5‐methyl‐2‐(1H)‐pyridone] (FD), a novel, low‐molecular‐weight pyridine agent, was developed and patented by the Pharmaceutical School of Central South University 17. Our previously reported data showed that FD exerts strong anti‐inflammatory and anti‐fibrotic effects on renal fibrosis and liver fibrosis 18, 19, 20. Meanwhile, FD could attenuate BLM‐induced experimental pulmonary inflammation, fibrosis and the protein expression of IL‐1β in BALFs in mice 21.

Hence, in this study, we explore mainly whether FD exerts its anti‐inflammatory and anti‐fibrotic effects through suppressing activation of NALP3 inflammasome and the IL‐1β/IL‐1R1/ MyD88/ NF‐κB signalling pathway *in vivo* and *in vitro*.

## Materials and methods

### Experimental animals and preparation

All animals used in this study were 6–8 weeks old male C57BL/6J mice (Experimental Animal Center of Central South University, Changsha, China). This study was carried out in strict accordance with the recommendations from the Guide for the Care and Use of Laboratory Animals published by the National Institutes of Health. The protocol was approved by the Medical Ethic Committee of the Xiangya Hospital of Central South University. Eighty C57BL/6J mice were randomly divided into five groups (each group includes sixteen mice): the control group (Control), the BLM group (BLM), the BLM/fluorofenidone group (FD), the BLM/YVAD‐fmk group (Cp1 inhibitor) and the BLM/Anakinet group (IL‐1Ra). The model of pulmonary fibrosis was established through intratracheally instillation with bleomycin (5 mg/kg) while Control group by instillation the same dose of normal saline. 24 hrs before establishment of the model, the FD group was fed with FD (500 mg/kg, once per day), the IL‐1Ra group was intraperitoneal injection with Anakinet (100 μg, twice per day). And the Cp1 inhibitor group was intraperitoneal injection with YVAD‐fmk (10 mg/kg) at 8 hrs and 4 hrs before the establishment of the model. Stochastically, eight mice in each group were killed separately on day 2 and day 14. The pathological section of the left lung tissues were harvested for hemotoxylin and eosin stain and Masson's trichrome stain, the right lung tissues were preserved by liquid nitrogen for ELISA, RT‐PCR and Western blotting analyses.

### Cell culture

The human actue monocytic leukaemia cell line THP‐1 and the RLE‐6TN (rat lung epithelial‐T‐antigen negative) cell line (provided by State Key Laboratory of Medical Genetics, Central South University, Hunan, China) were grown in Roswell Park Memorial Institute (RPMI) media or Ham's F12 medium supplemented with 10% foetal bovine serum at 37°C in 5% CO_2_. THP‐1 cells in suspension were differentiated into macrophage‐like cells by addition of Phorbol‐12‐myristate‐13‐acetate (PMA) (100 ng/ml) for 5 hrs, and then used in experiments. These THP‐1 cells were pre‐incubated with FD (2 mM) for 24 hrs or Cp1 inhibitor (YVAD‐fmk) for 1 hr and then exposed to 0.2 mg/ml monosodium urate (MSU) for 6 hrs. The protein of cell and cell supernatants were collected for Western blotting analyses or Co‐Immunoprecipitation. The RLE‐6TN cells were pre‐incubated with FD (2 mM) for 24 hrs, and then exposed to IL‐1β (10 ng/ml) for 1 hr or 30 min. The protein of cell and nuclear were collected for Western blotting analyses.

### Haematoxylin and eosin staining and Masson's Trichrome staining of lung

Lung tissues were collected and fixed in 4% paraformaldehyde, and then embedded in paraffin. Tissue section (4 μm) was stained with hemotoxylin and eosin and Masson's Trichrome, and the morphological changes were observed under light microscope. Microscopic fields (×100) greater than 8 is required to review each section, a numerical inflammatory score and a numerical fibrosis score ranging from 0 (normal) to 3 (more severe) were assigned using the criteria as Szapiel *et al*. described 22.

### Mediators measurements by ELISA

Interleukin‐1β, IL‐6, monocyte chemotactic protein‐1 (MCP‐1), myeloperoxidase (MPO) levels in lung homogenate were determined by ELISA, according to manufacturer's instructions (IL‐1β, R&D Systems Minneapolis, MN, USA; IL‐6, Merck Millipore, Bedford, MA, USA; MCP‐1, Uscnlife Science & Technology Company Houston, TX, USA; MPO, Uscnlife Science & Technology Company). The levels of IL‐1β, IL‐6, MCP‐1, MPO were calculated with reference to standard curves of purified recombinant IL‐1β, IL‐6, MCP‐1 or MPO at various dilutions.

### RNA extraction and real‐time PCR quantification

Total RNA was isolated from lung tissue of mice by Trizol Reagent according to the manufacturer's instructions (Invitrogen, Grand Island, NY, USA). The mRNA levels of α‐smooth muscle actin (SMA), fibronectin and collagen I were measured by real‐time PCR as described 21. The specific primers were designed from their GenBank sequences, were synthesized by Bio Basic (Generay Biotechnology, Shanghai, China), and are listed in Table 1.

**Table 1:** Nucleotide sequences of the primers used for real‐time PCR

Genes	Forward (5′–3′)	Reverse (5′–3′)
β‐Actin	GGCCAACCGTGAAAAGATGA	GACCAGAGGCATACAGGGACAA
α‐SMA	CTGAAGAGCATCCGACACTG	AGAGGCATAGAGGGACAGCA
Fibronectin	GAAGTCGCAAGGAAACAAGC	GTAGGTGAACGGGAGGACAC
Collagen I	GTCCTCCTGGTTCTCCTGGT	GACCGTTGAGTCCGTCTTTG

### Western blotting analysis

Lung tissues and the cells were lysed with a buffer [20 mM Tris‐HCL (pH 7.4), 4% sodium dodecyl sulphate (SDS), and 10% glycerol]. Lysates were boiled at 100°C for 10 min. The protein of cell supernatants was collected with the Amicon^®^ Ultra‐4 (Merck Millipore) according the protocol (Prod. No. AG‐CR1‐3951). The nuclear extract was prepared according to the manufacturer's instruction using NE‐PER Nuclear and Cytoplasmic Extracts Reagents kits (Pierce Biotechnology, Rockford, IL, USA). Protein concentrations were determined using the Bicinchoninic Acid (BCA) Protein Assay Kit (Pierce Biotechnology). For Western blot analysis, 20–40 μg of protein was separated on 10% or 12% SDS‐polyacrylamide gel under reducing conditions and transferred onto polyvinylidene difluoride membranes (Merck Millipore). Non‐specific antibody binding was blocked by pre‐incubation of the membranes in 1× Tris‐buffered‐saline containing 5% skim milk for 1 hr at room temperature. Membranes were then incubated overnight at 4°C with primary antibodies against α‐SMA (mouse monoclonal, 1:5000 dilution; Sigma‐Aldrich, St Louis, MO, USA), fibronectin (rabbit monoclonal, 1:2000 dilution; Santa Cruz Biotechnology, Dallas, TX, USA), IL‐1β (rabbit monoclonal, 1:400 dilution; Santa Cruz Biotechnology), caspase‐1 (rabbit monoclonal, 1:600 dilution; Santa Cruz Biotechnology), IL‐1R1 (rabbit monoclonal, 1:1000 dilution; Santa Cruz Biotechnology), MyD88 (rabbit monoclonal, 1:1000 dilution; Cell Signaling, Danvers, MA, United States), NALP3 (mouse monoclonal, 1:400 dilution; Adipogen, Waidmannstr, Hamburg, Germany), pro‐caspase‐1 (mouse monoclonal, 1:400 dilution; Santa Cruz Biotechnology), ASC (rabbit monoclonal; Santa Cruz Biotechnology), phospho‐IκBα (rabbit monoclonal, 1:1000 dilution; Cell Signaling), IκBα (rabbit monoclonal, 1:1000 dilution; Cell Signaling), phospho‐IKKα (rabbit monoclonal, 1:1000 dilution; Cell Signaling), inhibitor of nuclear factor kappa‐B kinase (IKKα) (rabbit monoclonal, 1:1000 dilution; Cell Signaling), PCNA (rabbit monoclonal, 1:1000 dilution; Cell Signaling) and β‐actin (mouse polyclonal, 1:5000; Sigma‐Aldrich). The secondary antibodies were a goat anti‐rabbit or goat antimouse horseradish peroxidase‐conjugated antibody (Sigma‐Aldrich).

### Flow cytometry

Reactive oxygen species generation was detected using the non‐fluorescent probe DCF‐DA (D6883; Sigma‐Aldrich). THP‐1 cells in suspension were differentiated into macrophage‐like cells by addition of PMA (100 ng/ml) for 6 hrs, and then used in experiments. These THP‐1 cells were pre‐incubated with FD (2 mM) for 1 hr and then exposed to 0.2 mg/ml MSU for 1 hr. These THP‐1 cells were incubated in 10 mM DCF‐DA for 30 min. at 37°C in the dark. Cells were washed with PBS for three times. And then, the cells were digested with the trypsin (no ethylenediaminetetraacetic acid). The level of mitochondrial ROS was detected in the FITC‐A channel of a FACScan flow cytometer, using 488‐nm excitation and 525‐nm emission. The number of stained cells was calculated using Cell Quest software (BD company).

### Co‐immunoprecipitation

To assess the protein composition and association of proteins in the inflammasome. RPMI cultures were lysed in 1 ml of Radio Immunoprecipitation Assay (RIPA) [1% NP‐40, 0.5% DOC (Deoxycholic acid; Sigma D6750; Sigma‐Aldrich), 0.1% SDS, 50 mM Tris.HCl, pH 8.0, 150 mM NaCl] with protease inhibitor mixture (Sigma‐Aldrich). About 2 mg of cell lysates were immunoprecipitated with 4 μg ASC‐antibody (rabbit monoclonal; Santa Cruz Biotechnology) and 50 μl protein G‐Agarose (p4691; Sigma‐Aldrich) in 4°C for 16 hrs with nutation, and then spin down the beads at 2.4 × *g* for 30 min. at 4°C. The pelleted beads were washed four times with PBS buffer (pH 7.3, 137 mM NaCl, 10 mM Na_2_HPO_4_, 1.8 mM KH_2_PO_4_). After the last wash, spin down once more at 5000 r.p.m. for 5 min. at 4°C without adding any buffer followed by aspirating the residual buffer. Add 15–20 μl SDS loading buffer mix the buffer and bead with finger tickling, and then boil the mixture for 10 min. and spin down with 14,000 r.p.m. for 10–30 sec. before analysis by Western Blotting using antibodies against NALP3 (mouse monoclonal, 1:400 dilution; Adipogen), pro‐caspase‐1 (mouse monoclonal, 1:400 dilution; Santa Cruz Biotechnology), and ASC (rabbit monoclonal, 1:400 dilution; Santa Cruz Biotechnology).

### Statistical analysis

All results are expressed as mean ± S.E. Using Star View ver. 5.0 software (SAS Institute, Cary, NC, USA), statistical differences among different groups were analysed using one‐way anova and *post hoc* multiple‐comparison tests. *P* < 0.05 was considered significant.

## Results

### Fluorofenidone attenuates BLM‐induced pulmonary inflammation at day 2

**Figure 1:**
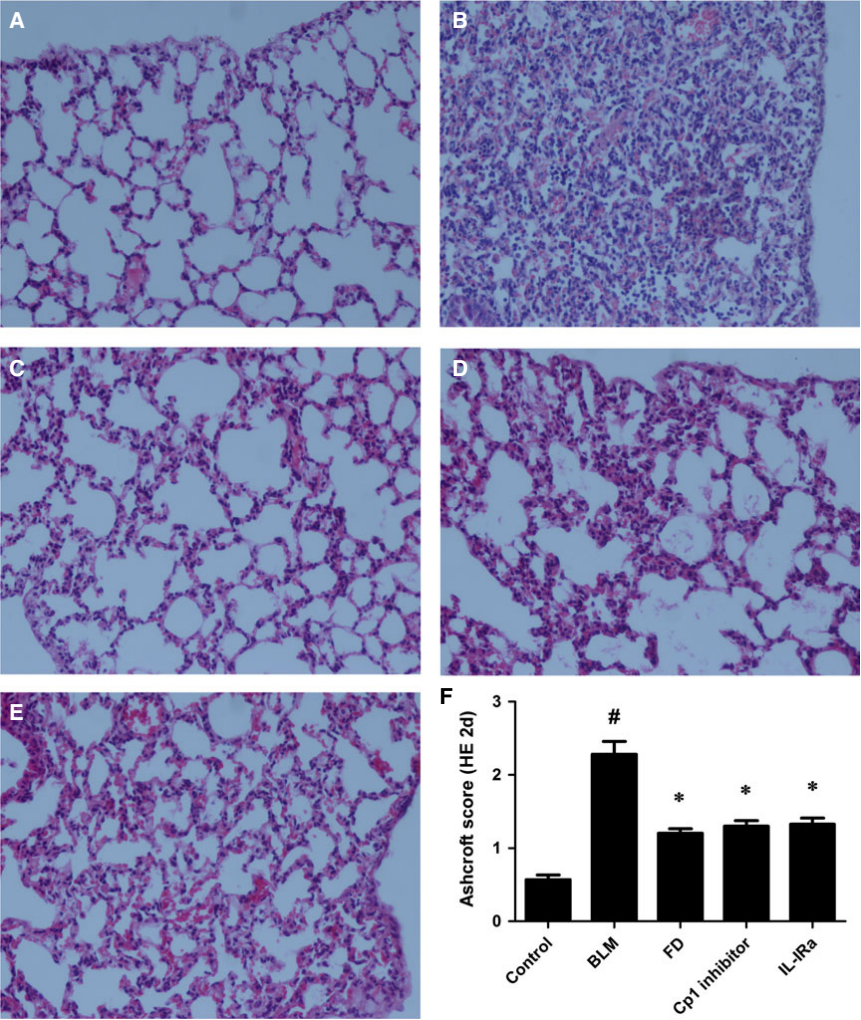
Fluorofenidone attenuates BLM‐induced pulmonary inflammation at day 2. The mice were intratracheally instillated with BLM and were administrated with FD, Cp1 inhibitor or IL‐1Ra. And animals were killed on the second day of BLM administration. Representative micrographs (hemotoxylin and eosin staining, 200×) of lungs from control mice (**A**), BLM mice (**B**), FD mice (**C**), Cp1 inhibitor mice (**D**) and IL‐1Ra mice (**E**) were showed. Quantitative assessment of pulmonary inflammation, as measured by Ashcroft histopathological scoring (**F**). Results are expressed as mean ± S.D., *n* = 8 mice per group, ^#^ *P* < 0.001 *versus* control groups, *P < 0.001 *versus* BLM groups.

We found that microscopic investigations on the second day of BLM administration showed severe alveolitis with abundant neutrophils in the alveoli, recruitment of mononuclear cells, destruction and repair with thickening of alveolar septa (Fig. 1B), compared with normal alveolar structure of control groups (Fig. 1A). Relative to the BLM groups, these changes were significantly reduced in the lungs of the FD, Cp1 inhibitor and IL‐1Ra groups (Fig. 1C–E).

The mean inflammatory score in the BLM group (1.850 ± 0.137) was markedly higher than the control group (0.574 ± 0.142; *P* < 0.001). Compared with the BLM group, administration of FD, Cp1 inhibitor or IL‐1Ra showed a significant reduction in the inflammatory score (*P* < 0.01 each; Fig. 1F).

### Fluorofenidone inhibits BLM‐induced IL‐1β, IL‐6, MCP‐1 and MPO increase in lung homogenate at day 2

**Figure 2:**
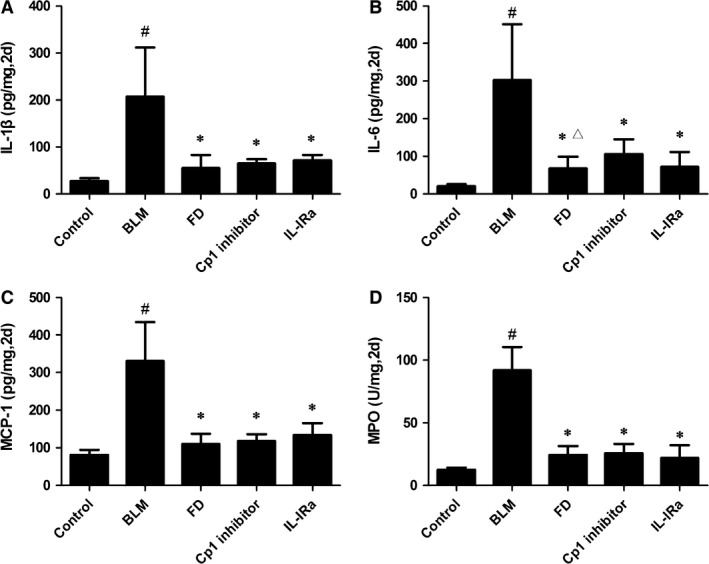
Fluorofenidone inhibits BLM‐induced the increase levels of IL‐1β, IL‐6, MCP‐1, MPO in lung homogenate at day 2. (**A**) IL‐1β, (**B**) IL‐6, (**C**) MCP‐1, (**D**) MPO concentrations in lung homogenates on the second day of BLM administration. Results are expressed as mean ± S.D., *n* = 5 mice per group, ^#^ *P* < 0.001 *versus* control groups, *P < 0.001 *versus* BLM groups, ^Δ^ *P* < 0.05 *versus* Cp1 inhibitor groups.

The acute lung injury induced by BLM administration led to severe inflammatory responses. On the second day of BLM administration, ELISA analysis showed that the inflammatory mediators IL‐1β (Fig. 2A) and IL‐6 (Fig. 2B), the MCP‐1 (Fig. 2C), the MPO activity (Fig. 2D) were significant increased than control group (*P* < 0.001). Compared with the BLM group, treatment with FD, Cp1 inhibitor or IL‐1Ra reduced the levels of IL‐1β, IL‐6, MCP‐1 and MPO in the lung (*P* < 0.01 each). And FD exerted stronger inhibitory effect than Cp1 inhibitor in IL‐6 expression (*P* < 0.05).

### Fluorofenidone inhibits BLM‐induced caspase‐1, IL‐1R1, MyD88 expressions in lung at day 2

**Figure 3:**
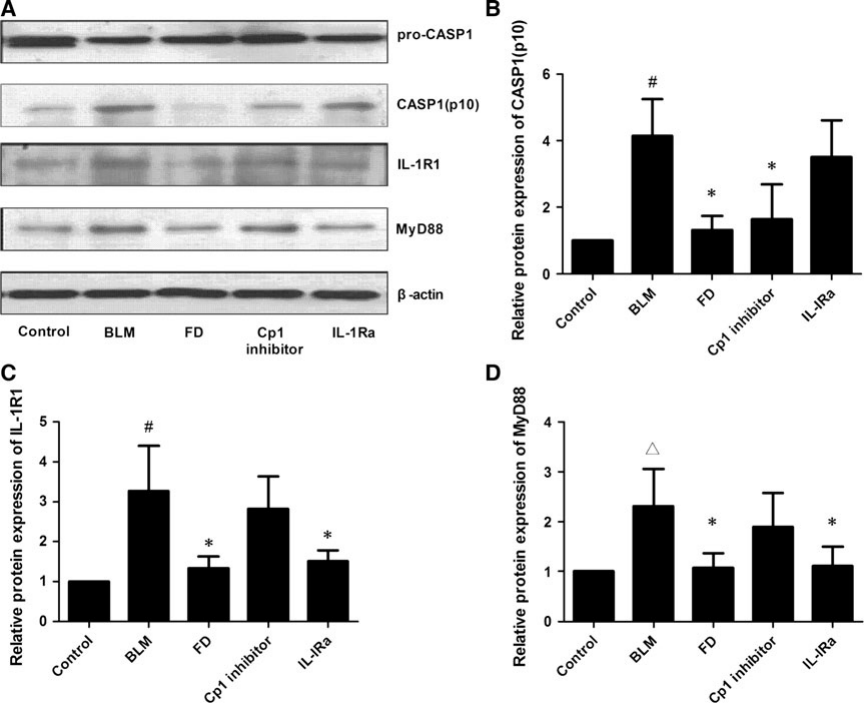
Fluorofenidone inhibits BLM‐induced caspase‐1, IL‐1R1, MyD88 expressions in lung at day 2. The mice were intra‐tracheally instillated with BLM and were administrated FD, Cp1 inhibitor or IL‐1Ra. And animals were killed on the second day of BLM administration. Total protein was isolated from the lungs. The protein levels of Caspase‐1, IL‐1R1, MyD88 were measured by Western blotting. (**A**) representative Western blot of Caspase‐1, IL‐1R1, MyD88, (**B**) quantitative analysis of active casepase1 (Casp1), (**C**) quantitative analysis of IL‐1R1, (**D**) quantitative analysis of MyD88. Results are expressed as mean ± S.D., *n* = 5 mice per group, ^#^ *P* < 0.001 *versus* control groups, ^∆^ *P* < 0.05 *versus* control groups, *P < 0.01 *versus* BLM groups.

As FD can attenuate BLM‐induced acute injury and inflammation in lung of mice, furthermore, we explored whether FD exerts its anti‐inflammatory effect through suppressing activation of NALP3 inflammasome and the IL‐1β/IL‐1R1/MyD88 signalling pathway. The expressions of caspase‐1, IL‐1R1 and MyD88 were detected by Western Blotting. We found that the protein expressions of active casepase‐1 (Casp1; *P* < 0.001), IL‐1R1 (*P* < 0.001) and MyD88 (*P* < 0.01) on the second day of BLM administration were markedly elevated in the lungs compared with control group (Fig. 3). And compared with BLM group, the level of Casp1 was significantly inhibited by FD or Cp1 inhibitor (*P* < 0.01 each), the expressions of IL‐1Rl, MyD88 were markedly inhibited by FD or IL‐1Ra (*P* < 0.01 each). Interestingly, we showed that compared with BLM group, IL‐1Ra did not inhibit the protein expression of Casp1 (*P* > 0.05), and Cp1 inhibitor did not inhibit the protein levels of IL‐1Rl and MyD88 (*P* > 0.05).

### Fluorofenidone attenuates BLM‐induced pulmonary inflammation and fibrosis at day 14

**Figure 4:**
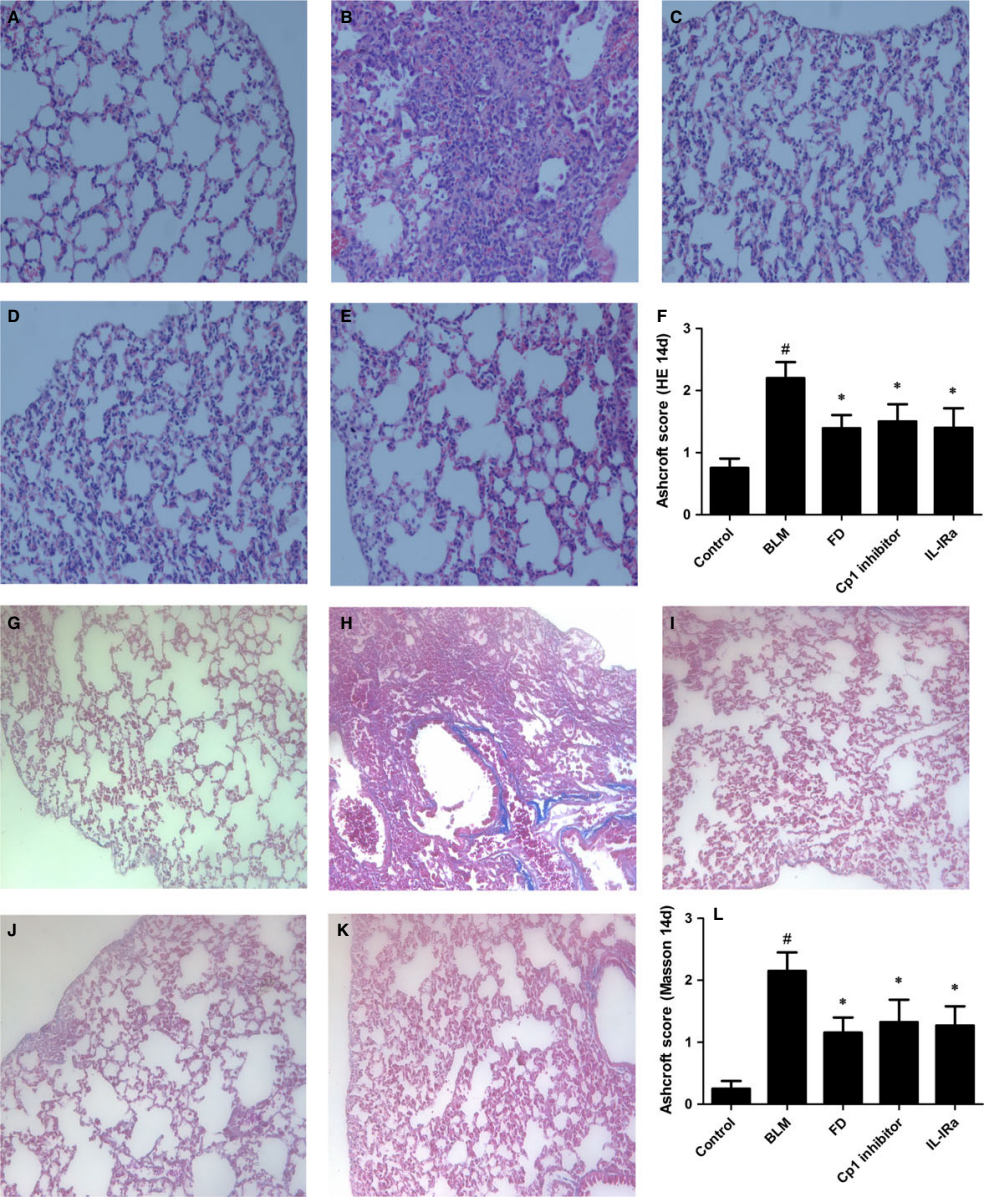
Fluorofenidone attenuates BLM‐induced pulmonary inflammation and fibrosis at day 14. The mice were intra‐tracheally instillated with BLM and were administrated FD, Cp1 inhibitor or IL‐1Ra. And animals were killed after 14 days. Representative micrographs (hemotoxylin and eosin staining, 200×) of lungs from control mice (**A**), BLM mice (**B**), FD mice (**C**), Cp1 inhibitor mice (**D**) and IL‐1Ra mice (**E**) were showed. Quantitative assessment of pulmonary inflammation, as measured by Ashcroft histopathological scoring (**F**). Representative micrographs (Masson's Trichrome staining, 200×) of lungs from control mice (**G**), BLM mice (**H**), FD mice (**I**), Cp1 inhibitor mice (**J**) and IL‐1Ra mice (**K**) were showed. Quantitative assessment of pulmonary fibrosis, as measured by Ashcroft histopathological scoring (**L**). Results are expressed as mean ± S.D., *n* = 7 mice in control BLM or FD groups, *n* = 6 mice in Cp1 inhibitor or IL‐1Ra groups, ^#^ *P* < 0.001 *versus* control groups, *P < 0.001 *versus* BLM groups.

Microscopic investigations showed that, compared with control group (Fig. 4A and G), the alveolar developed severe and extensive inflammation and fibrosis at day 14 after BLM administration (Fig. 4B and H), with distorted pulmonary architecture, massive infiltration of leucocytes and excessive deposition of collagen in the interstitium. Relative to BLM group, the lungs from FD, Cp1 inhibitor or IL‐1Ra groups exhibited significant reduction in inflammation and fibrosis (Fig. 4C–E and I–K).

The mean inflammatory score (2.200 ± 0.259) in BLM group was markedly increased than control group (0.750 ± 0.153; *P* < 0.001; Fig. 4F). And the mean fibrosis score (2.150 ± 0.298) in BLM group was also higher than control group (0.250 ± 0.125; *P* < 0.001; Fig. 4L). Compared with BLM group, administration of FD, Cp1 inhibitor or IL‐1Ra showed a significant reduction in inflammatory score as well as fibrosis score (*P* < 0.001 each).

### Fluorofenidone inhibits BLM‐induced IL‐1β, IL‐6, MCP‐1 and MPO increase in lung homogenate at day 14

**Figure 5:**
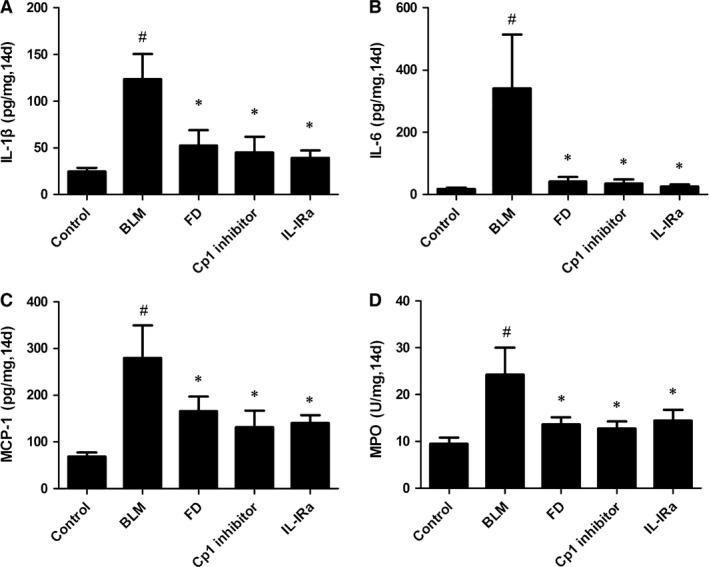
Fluorofenidone inhibits BLM‐induced IL‐1β, IL‐6, MCP‐1, MPO mRNA expressions in lung at day 14. (**A**) IL‐1β, (**B**) IL‐6, (**C**) MCP‐1, (**D**) MPO concentrations in lung homogenates 14 days after intra‐tracheally instillation with BLM. Results are expressed as mean ± S.D., *n* = 5 mice per group, ^#^ *P* < 0.001 *versus* control groups, *P < 0.05 *versus* BLM groups.

The sustained inflammation induced by BLM administration led to pulmonary fibrosis. We found that the levels of IL‐1β (Fig. 5A), IL‐6 (Fig. 5B), MCP‐1 (Fig. 5C) and MPO (Fig. 5D) at day 14 after BLM administration were significantly elevated than control group (*P* < 0.001). Compared with BLM group, treatment with FD, Cp1 inhibitor or IL‐1Ra reduced the levels of IL‐1β, IL‐6, MCP‐1 and MPO in the lung homogenate (*P* < 0.01 each).

### Fluorofenidone down‐regulates BLM‐induced expressions of α‐SMA, fibronectin and collagen I in lung at day 14

**Figure 6:**
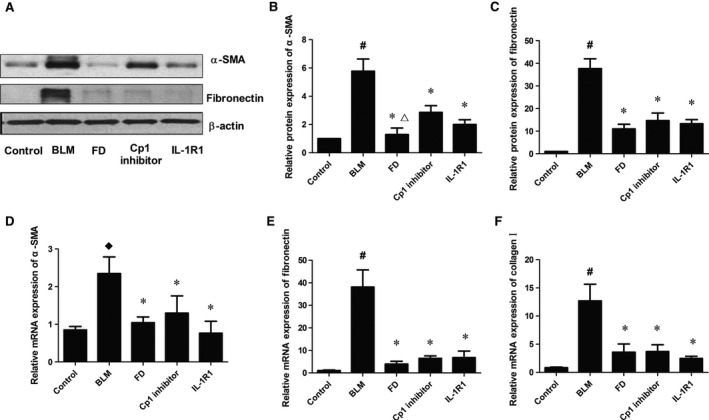
Fluorofenidone down‐regulates BLM‐induced expressions of α‐SMA, fibronectin and collagen I in lung at day 14. The mice were intra‐tracheally instillated with BLM and were administrated FD, Cp1 inhibitor or IL‐1Ra. And animals were killed after 14 days. Total protein and mRNA were isolated from the lungs. The expressions of α‐SMA, fibronectin, collagen I were measured by Western blotting and/or real‐time PCR. (**A**) representative Western blot of α‐SMA and fibronectin, (**B**) quantitative analysis of α‐SMA, (**C**) quantitative analysis of fibronectin, (**D**) mRNA expression of α‐SMA, (**E**) mRNA expression of fibronectin, (**F**) mRNA expression of collagen I. Results are expressed as mean ± S.D., *n* = 5 mice per group, ^#^ *P* < 0.001 *versus* control groups, ^♢^ *P* < 0.05 *versus* control groups, *P < 0.05 *versus* BLM groups, ^∆^ *P* < 0.05 *versus* Cp1 inhibitor groups.

The protein expressions of α‐SMA and fibronectin at day 14 after treatment with BLM were 5.5‐ and 40‐fold higher in the lungs compared with control group, respectively (*P* < 0.01). Furthermore, the expressions of α‐SMA and fibronectin were markedly inhibited by FD, Cp1 inhibitor or IL‐1Ra (*P* < 0.05 each; Fig. 6A–C), and FD showed stronger inhibitory effect than Cp1 inhibitor (*P* < 0.05).

Real‐time PCR analysis showed that the mRNA levels of α‐SMA (*P* < 0.05), fibronectin (*P* < 0.01), and collagen I (*P* < 0.01) were markedly elevated at day 14 after treatment with BLM compared with control group (Fig. 6D–F). Compared with BLM group, treatment with FD, Cp1 inhibitor or IL‐1Ra reduced the mRNA levels of α‐SMA, fibronectin and collagen I in the lung (*P* < 0.05 each).

### Fluorofenidone inhibits BLM‐induced caspase‐1, IL‐1R1 and MyD88 expressions in lung at day 14

**Figure 7:**
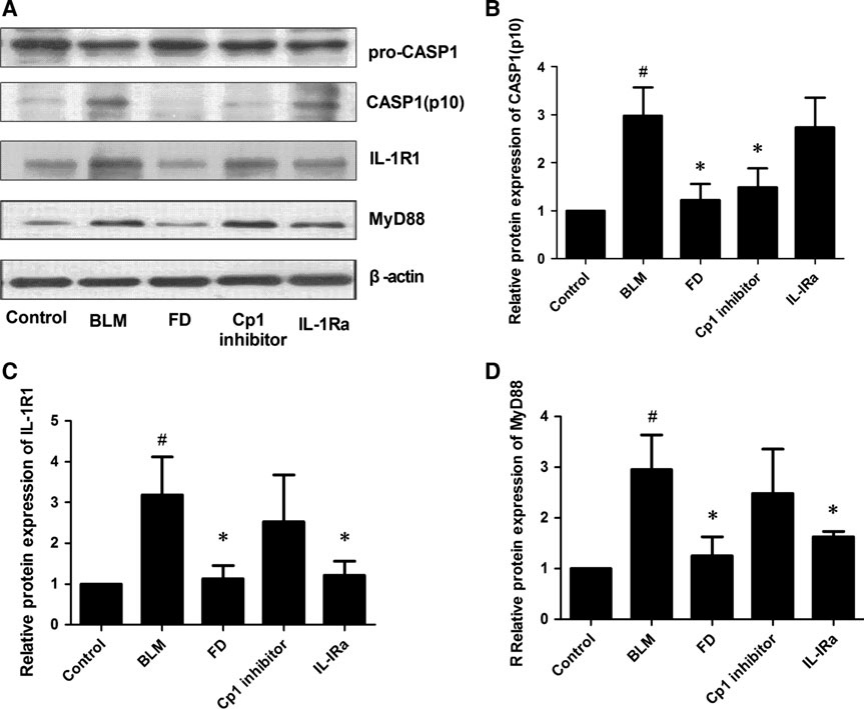
Fluorofenidone inhibits BLM‐induced caspase‐1, IL‐1R1, MyD88 expressions in lung at day 14. The mice were intra‐tracheally instillated with BLM and were administrated FD, Cp1 inhibitor or IL‐1Ra. And animals were killed after 14 days. Total protein was isolated from the lungs. The protein levels of Caspase‐1, IL‐1R1, MyD88 were measured by Western blotting. (**A**) representative Western blot of Caspase‐1, IL‐1R1, MyD88, (**B**) quantitative analysis of active casepase1 (Casp1), (**C**) quantitative analysis of IL‐1R1, (**D**) quantitative analysis of MyD88. Results are expressed as mean ± S.D., *n* = 5 mice per group, ^#^ *P* < 0.001 *versus* control groups, *P < 0.01 *versus* BLM groups.

The protein expressions of active Casp1, IL‐1R1 and MyD88 at day 14 after treated with BLM were significantly increased in the lung compared with control group (*P* < 0.001; Fig. 7). And compared with BLM group, the level of Casp1 was significantly inhibited by FD or Cp1 inhibitor (*P* < 0.01 each), and the expressions of IL‐1Rl and MyD88 were markedly inhibited by FD or IL‐1Ra (*P* < 0.01 each). Interestingly, we also found that compared with BLM group, IL‐1Ra did not inhibit the protein expression of Casp1 (*P* > 0.05), and Cp1 inhibitor did not inhibit the protein levels of IL‐1Rl or MyD88 (*P* > 0.05).

### Fluorofenidone inhibits MSU‐induced protein expressions of caspase‐1 and IL‐1β in THP‐1 cells

**Figure 8:**
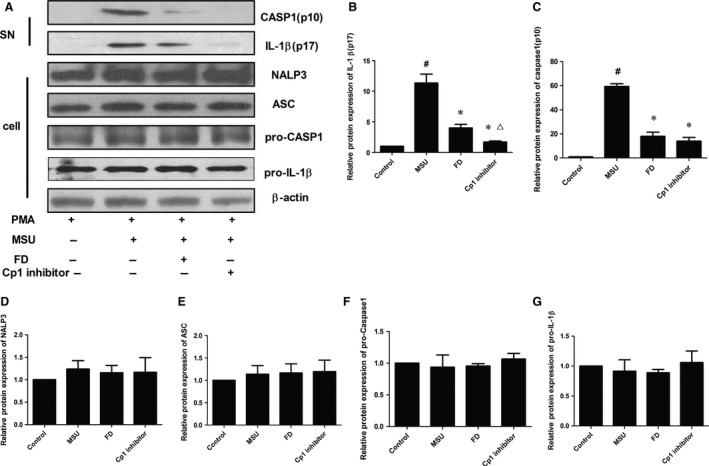
Fluorofenidone inhibits MSU‐induced protein expressions of caspase‐1 and IL‐1β in THP‐1 cells. The THP‐1 cells were pre‐incubated with FD or Cp1 inhibitor, and then exposed to MSU. The protein of cell and cell supernatants were collected. The protein expressions of IL‐1β, Pro‐IL‐1β, caspase‐1, NALP3, ASC and Pro‐caspase‐1 were measured by Western blotting. (**A**) representative Western blot of IL‐1β, pro‐IL‐1β, caspase‐1 and pro‐caspase‐1, NALP3 and ASC, (**B**) quantitative analysis of IL‐1β, (**C**) quantitative analysis of caspase‐1, (**D**) quantitative analysis of NALP3, (**E**) quantitative analysis of ASC, (**F**) quantitative analysis of pro‐caspase‐1 (**G**) quantitative analysis of Pro‐IL‐1β. Results are expressed as mean ± S.D., *n* = 3 per group, ^#^ *P* < 0.01 *versus* control groups, *P < 0.01 *versus* MSU groups, ^∆^ *P* < 0.01 *versus* FD groups.

It is reported that uric acid is a danger signal activating NALP3 inflammasome in BLM‐induced lung injury inflammation and fibrosis 11. Furthermore we examined whether FD could inhibit MSU‐induced activation of inflammasome *in vitro*. Compared with control group, the levels of IL‐1β (p17) and caspase‐1 (p10) were 11.3‐ and 60.1‐fold higher in THP‐1 cells culture supernatant after treated with MSU (*P* < 0.01; Fig. 8A–C). And pre‐treated with FD for 24 hrs or Cp1 inhibitor for 1 hr ameliorated the protein expressions of IL‐1β (p17) and caspase‐1 (p10; *P* < 0.01 with MSU groups). Cp1 inhibitor had stronger inhibitory effect than FD in IL‐1β (p17) protein expression after being treated with MSU (*P* < 0.01). While the protein expressions of NALP3, ASC, Pro‐caspase‐1 and Pro‐IL‐1β were not influenced by MSU, FD or Cp1 inhibitor in THP‐1 cells (*P* > 0.05 each; Fig. 8A, D, E, F and G).

### Fluorofenidone inhibits MSU‐induced interation of NALP3 inflammasome‐associated molecules in THP‐1 cells

**Figure 9:**
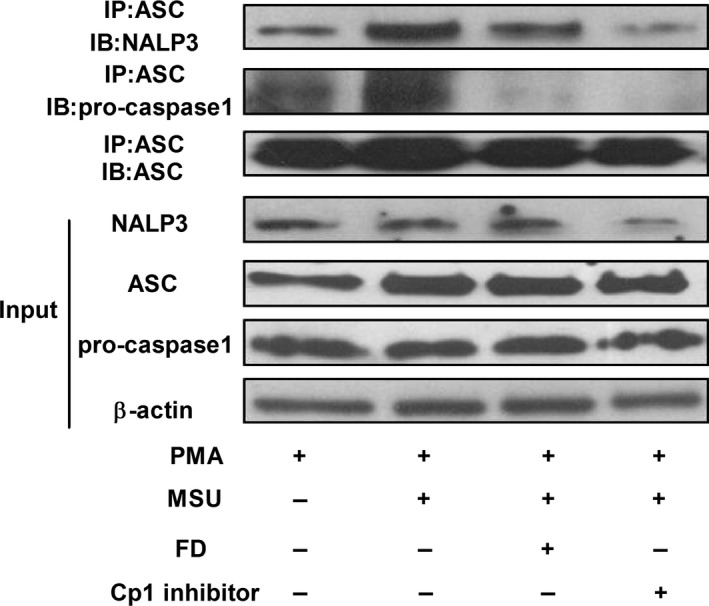
Fluorofenidone inhibits MSU‐induced the molecular interactions between NALP3 and ASC, and between ASC and pro‐caspase‐1. The THP‐1 cells were pre‐incubated with FD and Cp1 inhibitor, and exposed to MSU. Cell lysates were analysed for interaction of NALP3 and ASC, ASC and ASC, ASC and pro‐caspase‐1 by Immunoprecipitation and Western blotting. Expressions of NALP3, ASC, pro‐caspase‐1and β‐actin were analysed by Western blotting using the identical total cell lysates. Data are representative of three experiments.

It is reported that once inflammasome activated, the nucleotide‐binding oligomerization domain‐like receptors (NLRs) will aggregate with the adaptor protein ASC to serve as a scaffold for the assembly of the inflammasome 23. Assembly of the inflammasome activates caspase1, which then participates in substrate cleavage of pro‐IL‐1β into its active forms, inducing further secretion from the cell. To explore whether FD inhibited the activation of inflammasome through inhibiting the interaction of NALP3 inflammasome‐associated molecules. We found that MSU enhanced the molecular interactions between ASC and NALP3 or pro‐caspase‐1 compared with control group (Fig. 9). And, interestingly, FD or Cp1 inhibitor pre‐treatment inhibited MSU‐induced interaction between ASC and NALP3 or pro‐caspase‐1.

### Fluorofenidone inhibits MSU‐induced intracellular ROS production in THP‐1 cells

**Figure 10:**
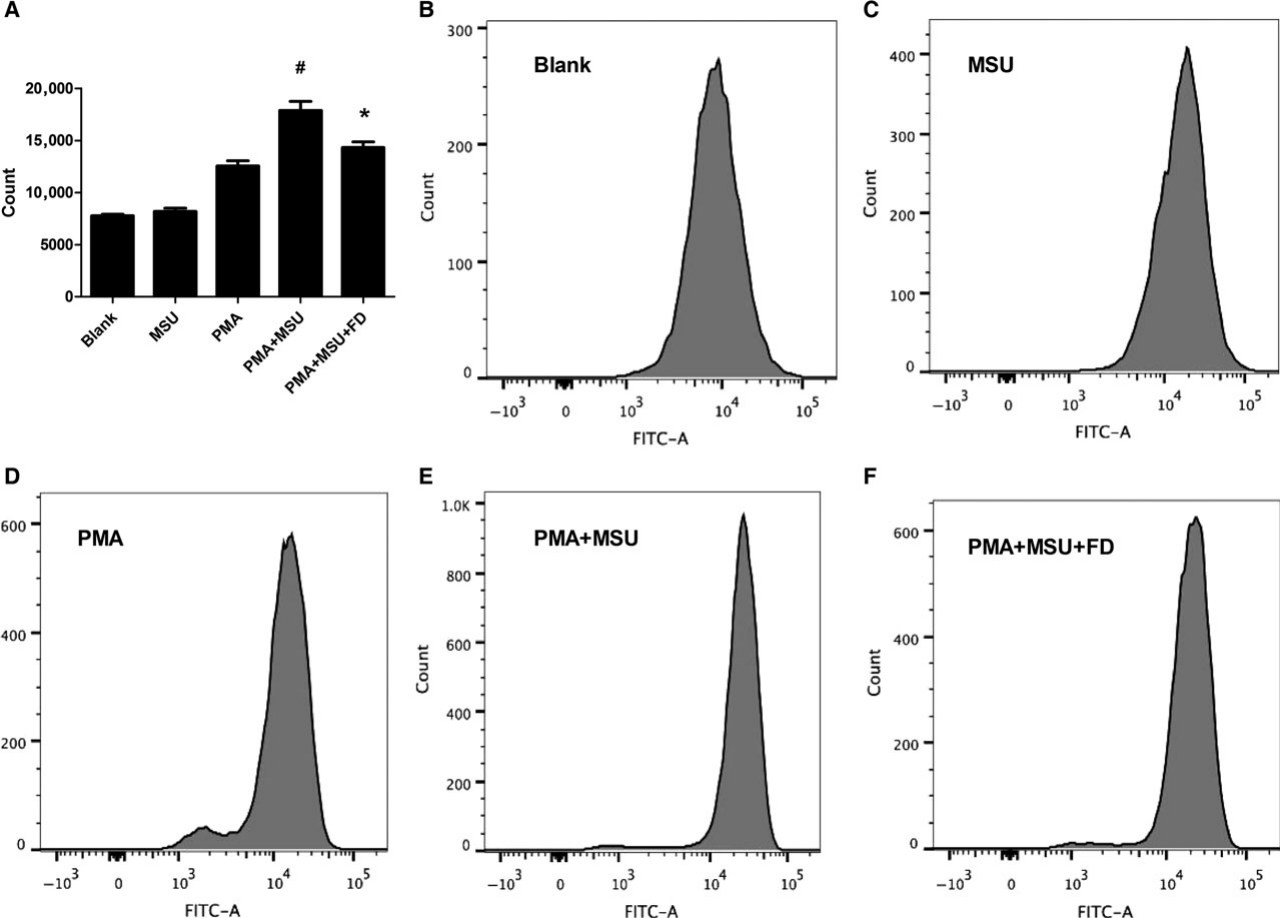
Fluorofenidone inhibits MSU‐induced intracellular ROS production in THP‐1 cells. The THP‐1 cells were pre‐incubated with FD, and then exposed to MSU. These THP‐1 cells were incubated in 10 mM DCF‐DA for 30 min. at 37°C in the dark. ROS generation was detected using the non‐fluorescent probe DCF‐DA (**A**) ROS production was detected *via* staining with 10 mM DCF‐DA, (**B**–**F**) ROS production was analysed by flow cytometry. Results are expressed as mean ± S.D., *n* = 3 per group, ^#^ *P* < 0.05 *versus* PMA groups, *P < 0.05 *versus* PMA+MSU groups.

Reactive oxygen species has been proposed as regulatory factors of the NLRP3 inflammasome 24, 25. After treatment with 0.2 mg/ml MSU, the level of ROS was significantly (*P* < 0.05) high in THP‐1 cells (Fig. 10A). And FD pre‐treatment inhibited MSU‐induced intracellular ROS production (*P* < 0.05).

### Fluorofenidone has no effects on IL‐1β‐induced protein expression of IL‐1R1, MyD88, p‐IKKα, p‐IκB‐α, inhibits MSU‐induced NF‐кB nuclear translocation in RLE‐6TN cells

**Figure 11:**
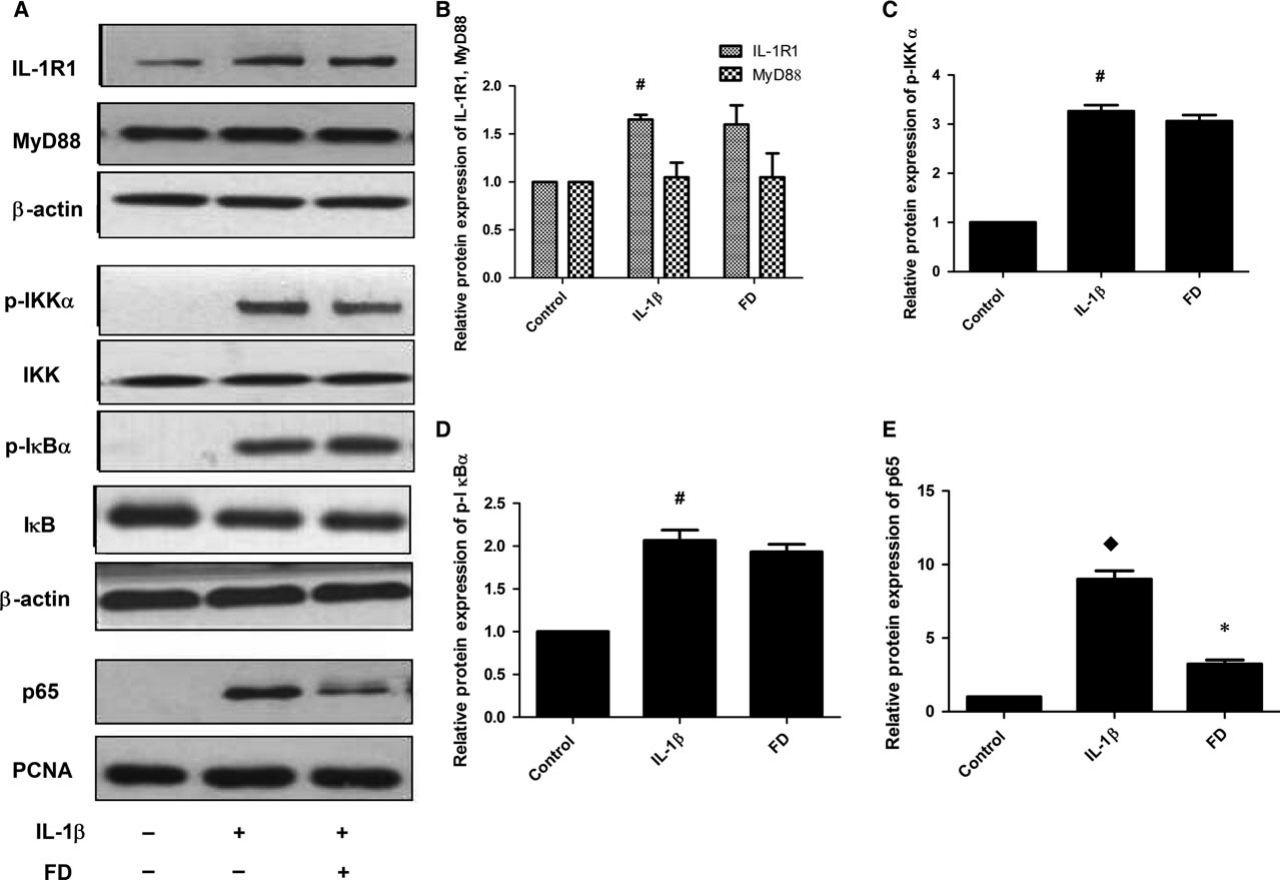
Fluorofenidone has no effects on IL‐1β‐induced protein expression of IL‐1R1, MyD88, p‐IKKα, p‐IκB‐α, Inhibits MSU‐induced NF‐кB nuclear translocation in RLE‐6TN cells. The RLE‐6TN cells were pre‐incubated with FD, and then exposed to IL‐1β. The protein levels of IL‐1R1, MyD88, p‐ IKKα, p‐IκB‐α and the nuclear protein p65 were measured by Western blotting. (**A**) representative Western blot of IL‐1R1, MyD88, p‐IKKα, p‐IκB‐α and p65 (**B**) quantitative analysis of IL‐1R1, MyD88, (**C**) quantitative analysis of p‐IKKα, (**D**) quantitative analysis of p‐IκB‐α, (**E**) quantitative analysis of p65. Results are expressed as mean ± S.D., *n* = 3 per group, ^#^ *P* < 0.05 *versus* control groups, ^♦^ *P* < 0.01 *versus* control groups, *P < 0.01 *versus* IL‐1β groups.

As a central pro‐inflammatory mediator, IL‐1β leads to the activation of IL‐1R1/MyD88 signalling pathways, which induces the activation of transcription factor NF‐кB. To determine whether FD could inhibit IL‐1β‐induced activation of the IL‐1β/IL‐1R1/MyD88/NF‐кB pathway, we first examined the effects of FD on IL‐1β‐induced the expressions of IL‐1R1 and MyD88. Interleukin‐1β stimulation significantly increased the expression of IL‐1R1 (*P* < 0.05), FD did not inhibit the protein level of IL‐1Rl (Fig. 11A and B). Interleukin‐1β or FD had no effects on the expression of MyD88 (Fig. 11A and B). Next, we tested the effects of FD on IL‐1β‐induced phosphorylation of IKKα and IκB‐α, the upstream activating signal for the NF‐кB nuclear translocation. Interleukin‐1β stimulation significantly increased the phosphorylation of IKKα and IκB‐α (*P* < 0.05), but addition of FD did not affect the phosphorylation of IKKα or IκB‐α induced by IL‐1β (Fig. 11A, C and D). Furthermore, we explored whether FD could inhibit IL‐1β‐induced NF‐кB nuclear translocation. Interestingly, FD treatment markedly blocked the accumulation of p65 in the nucleus induced by IL‐1β (*P* < 0.05; Fig. 11A and E).

## Discussion

Idiopathic pulmonary fibrosis is the interstitial pneumonia with the worst prognosis—the mortality rate at 3–5 years after diagnosis is 50% 1, 26. Pirfenidone is now approved for the treatment of pulmonary fibrosis and exerts anti‐inflammatory and anti‐hydroxyl radical activities 26. Fluorofenidone, a novel, low‐molecular‐weight pyridine agent, has a chemical structure similar to that of pirfenidone and was developed and patented by the Pharmaceutical School of Central South University 17. Previous data showed that FD exerts a stronger anti‐inflammatory and anti‐fibrotic effect than PFD on the model of pulmonary fibrosis 21, while its pharmacology mechanisms are needed to be further elucidated. The activation of inflammasome and IL‐1R1/MyD88 signalling pathway plays an important role in the pathogenesis of idiopathic pulmonary fibrosis 8, 11, 12. Here, we present evidence that FD can attenuate BLM‐induced pulmonary inflammation and fibrosis through suppressing activation of NALP3 inflammasome and may abrogate IL‐1β/IL‐1R1/MyD88/NF‐κB signalling pathway *in vivo* and *in vitro*.

Interleukin‐1β is implicated in the pathogenesis of idiopathic pulmonary fibrosis. *In vivo*, transient overexpression of IL‐1β in airway epithelial cells promoted the release of the pro‐inflammatory cytokines IL‐6 and TNF‐α, followed by a significant increase in TGF‐β1 and platelet derived growth factor that induced the deposition of collagen in the lung 27.

Interleukin‐1β production requires two signalling pathways. The first one is *via* NALP3 inflammasome activation, which is characterized by cleaving caspase‐1. The inflammasomes are pattern recognition receptors that are capable of recognizing a diverse range of conserved molecular motifs unique to microorganisms. In addition, they can detect chemical alarm signals produced by activated cells in host infection and tissue damage 28. Many studies suggest that several mediators, such as uric acid, ATP, high mobility group box1 protein, produced after lung damage leading to the activation of NALP3 inflammasome, followed by production of IL‐1β, inflammation, remodeling, and fibrosis. In our study, we found that stimulation with BLM led to severe alveolitis with abundant inflammation cells infiltration, excessive deposition of collagen in the interstitium, and increased production of IL‐1β, IL‐6, MCP‐1, MPO, α‐SMA, Fibronectin and Collagen I. Administration of FD attenuated BLM‐induced early and late pulmonary inflammation and production of IL‐1β, IL‐6, MCP‐1, MPO, as well as pulmonary fibrosis and the expressions of α‐SMA, Fibronectin and Collagen I. To further explore the mechanism, YVAD‐fmk (Cp1 inhibitor) or Anakinet (IL‐1Ra) was used to treat the mice stimulated with BLM, and we found that Cp1 inhibitor and IL‐1Ra also attenuated BLM‐induced pulmonary inflammation and fibrosis. These results are consistent with other reports by Gasse *et al*., who demonstrated specific blockade of the caspase‐1 activation by z‐YVAD‐fmk and specific blockade of IL‐1R1 by IL‐1 receptor antagonist dramatically reduced BLM‐induced lung inflammation 8, 11. Interestingly, Western Blotting showed that BLM‐induced increased protein expression of Casp1 (p10), administration of FD or Cp1 inhibitor inhibited the maturation of caspase‐1. BLM‐induced uric acid released from injured lung cells constitute major endogenous danger signals 11, and exogenous uric acid crystals have been shown to activate the NALP3 inflammasome, leading to IL‐1β–dependent inflammation in the peritoneal cavity 29, 30. We found that in THP‐1 cells MSU enhanced the molecular interactions between ASC and NALP3 or pro‐caspase‐1, leading to pro‐caspase‐1 activation and IL‐1β maturation. And treatment with FD or Cp1 inhibitor attenuated this effect of MSU. Oxidative stress is crucial for NLRP3 inflammasome activation 24, 31. Generation of ROS through MSU crystals binding to cell surfaces was found to be a potential mediator in NLRP3 inflammasome activation 32. Flow Cytometry showed that in THP‐1 cells ROS generation was triggered by MSU, and treatment with FD attenuated this effect of MSU. These data demonstrated that FD attenuates BLM‐induced pulmonary inflammation and fibrosis through suppressing activation of NALP3 inflammasome.

Second, IL‐1β/IL‐1R1/MyD88/NF‐κB pathway also affects the production of IL‐1β. Interleukin‐1β binds to IL‐1 receptor, initiating the recruitment of MyD88. Once recruited to the IL‐1R1 complex, MyD88 activates the IL receptor‐associated kinases 1–4 (IRAK1 to 4), which become phosphorylated and dissociate from MyD88 and associate with the tumour necrosis factor receptor‐associated factor, leading to the activation of IKK and the activation of IкB, which induces the activation of transcription factor NF‐κB 10, 33, results in mRNA expression of IL‐1β. Exogenous IL‐1β induces acute lung inflammation and lung tissue remodeling, progressing to fibrosis 8. Interleukin‐1R1 or MyD88 deficient mice, or administration of Anakinet abrogated inflammation and fibrosis induced by BLM 8. In our study, we found that BLM increased protein expressions of IL‐1R1 and MyD88, administration of FD or IL‐1Ra inhibited the levels of IL‐1R1 and MyD88, while Cp1 inhibitor did not. Interleukin‐1R1 initiates and amplifies the inflammatory response upon binding to the agonist ligands IL‐1α and IL‐1β, and is inhibited upon binding to the antagonist ligand IL‐1Ra 34, but Cp1 inhibitor inhibited the activation of NALP3 inflammasome only leading to attenuated IL‐1β maturation. *In vitro*, we found that IL‐1β stimulation significantly increased the expression of IL‐1R1 in RLE‐6TN cells, FD did not inhibit the protein level of IL‐1R1. And IL‐1β or FD had no effects on the protein expression of MyD88. Furthermore, we found that FD effectively inhibited IL‐1β–induced NF‐кB (p65) nuclear translocation. Interestingly, FD had no effect on the phosphorylation of IKKα and IκB‐α induced by IL‐1β, suggesting that FD inhibited the NF‐кB pathway by blocking NF‐кB nuclear translocation rather than inhibition of the phosphorylation of IKKα or IκB‐α, which are the upstream activating signal for the NF‐кB nuclear translocation. These data demonstrated that FD may attenuate BLM‐induced pulmonary inflammation and fibrosis through suppressing the IL‐1β/IL‐1R1/MyD88/NF‐κB signalling pathway, further studies should be conducted to identify it.

## Conclusion

In conclusion, FD attenuates BLM‐induced pulmonary inflammation and fibrosis through suppressing the activation of NALP3 inflammasome and the IL‐1β/IL‐1R1/MyD88/NF‐κB signalling pathway. However, the pharmacology mechanism of FD and its direct molecular target are not well understood, further studies should be conducted to identify it.

## Conflicts of interest

The authors confirm that there are no conflicts of interest.
